# Acute exacerbation of idiopathic pulmonary fibrosis induced by pertussis: the first case report

**DOI:** 10.1186/s12890-019-0779-9

**Published:** 2019-01-14

**Authors:** Kuniaki Hirai, Tetsuya Homma, Fumihiro Yamaguchi, Munehiro Yamaguchi, Shintaro Suzuki, Akihiko Tanaka, Tsukasa Ohnishi, Hironori Sagara

**Affiliations:** Department of Internal Medicine, Division of Allergology and Respiratory Medicine, Showa University School of Medicine, 1-5-8 Hatanodai, Shinagawa-ku, Tokyo, Japan

**Keywords:** Acute exacerbation, Idiopathic interstitial pneumonia, Idiopathic pulmonary fibrosis, Pertussis, Whooping cough

## Abstract

**Background:**

Acute exacerbation of idiopathic pulmonary fibrosis (AE-IPF) is a severe condition with limited treatment strategies. Although respiratory infection is a major cause of AE-IPF, no reports have indicated pertussis infection as a cause. Here we report two cases of pertussis infection-induced AE-IPF.

**Case presentation:**

Both patients presented with a chief complaint of acute respiratory distress and were previously diagnosed with idiopathic pulmonary fibrosis (IPF). Neither patient had received any pertussis vaccination since adolescence. Both patients were diagnosed with AE-IPF accompanying acute pertussis infection based on chest computed tomography and serum pertussis toxin antibody > 100 EU/mL. Both patients were treated with macrolide antibiotics and systemic corticosteroids. Both patients were able to be discharged and return home.

**Conclusions:**

The presence of pertussis infection in AE-IPF can present a diagnostic challenge, as coughing accompanying pertussis may be difficult to distinguish from IPF-associated coughing. Pertussis infection should be assayed in AE-IPF patients. Since pertussis can be prevented with vaccination and is expected to be affected by antibiotics, consideration of pertussis infection as a causative virulent factor of AE-IPF may be important for management of subjects with IPF.

## Background

Idiopathic pulmonary fibrosis (IPF) normally follows a chronic and progressive course. Respiratory failure that occurs during the course of this disease is known as acute exacerbation of IPF (AE-IPF), which may be caused by infection [1]. The majority of published studies investigating the causes of acute exacerbation of IPF have been primarily focused on viral sources of infection, rather than bacterial sources [2]. To our knowledge, there have been no previous reports of pertussis as a causative factor of AE-IPF. Here, we report our experience in managing two cases of AE-IPF that have been induced by acute pertussis infection. Written informed consent was obtained from the participant for the publication of this case report. This case report was written in accordance with the Declaration of Helsinki and its publication was approved by our University Ethics Committee (approval number, 2616).

## Case presentation

**Fig. 1:**
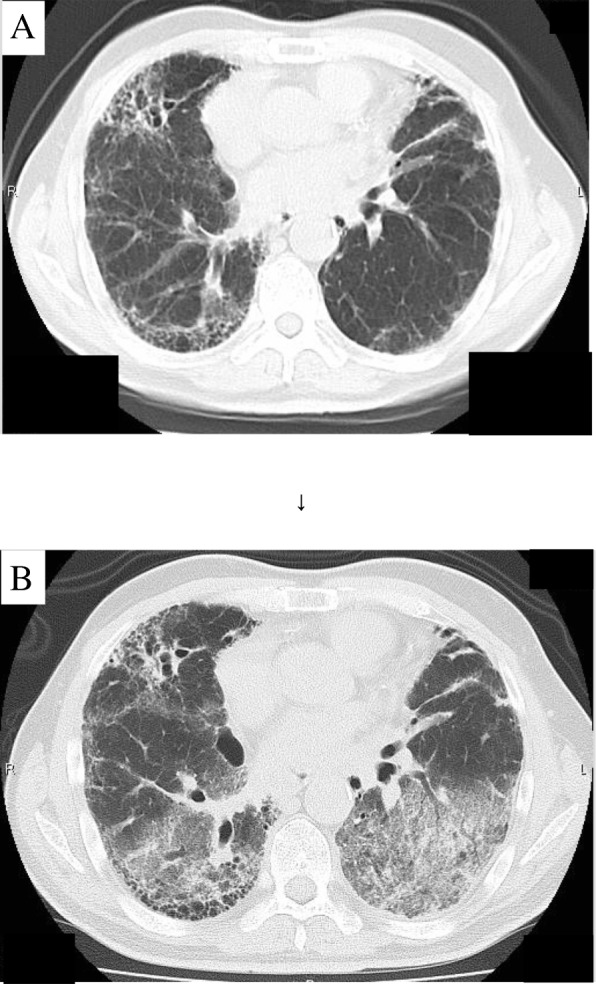
Radiological findings. Chest CT findings for a 69-year-old man. **a** Chest CT findings at 3 weeks before admission. **b** Chest CT findings at the time of admission showed new ground-glass opacity findings over a broad range of bilateral lung fields

Case 1: The patient was a 69-year-old man who was diagnosed with IPF 5 years prior to the current episode. He complained of respiratory distress during exertion and dry cough without any treatment. Physical examination revealed bilateral fine crackles in the lung. The patient was admitted to our hospital because of a sudden worsening of his respiratory distress and was diagnosed with AE-IPF based on a poor blood oxygen concentration and the observation of new ground-glass opacity findings over a broad range of bilateral lung fields during computed tomography (CT) scanning (Fig. 1). A high level of pertussis toxin (PT) antibodies (147 EU/mL) was noted in samples taken on day 1 of admission. After successful life-saving treatment, the PT level decreased to 52 EU/mL, as measured 30 days after admission. The patient began long-term oxygen therapy (LTOT) and was then discharged to his home.

**Fig. 2:**
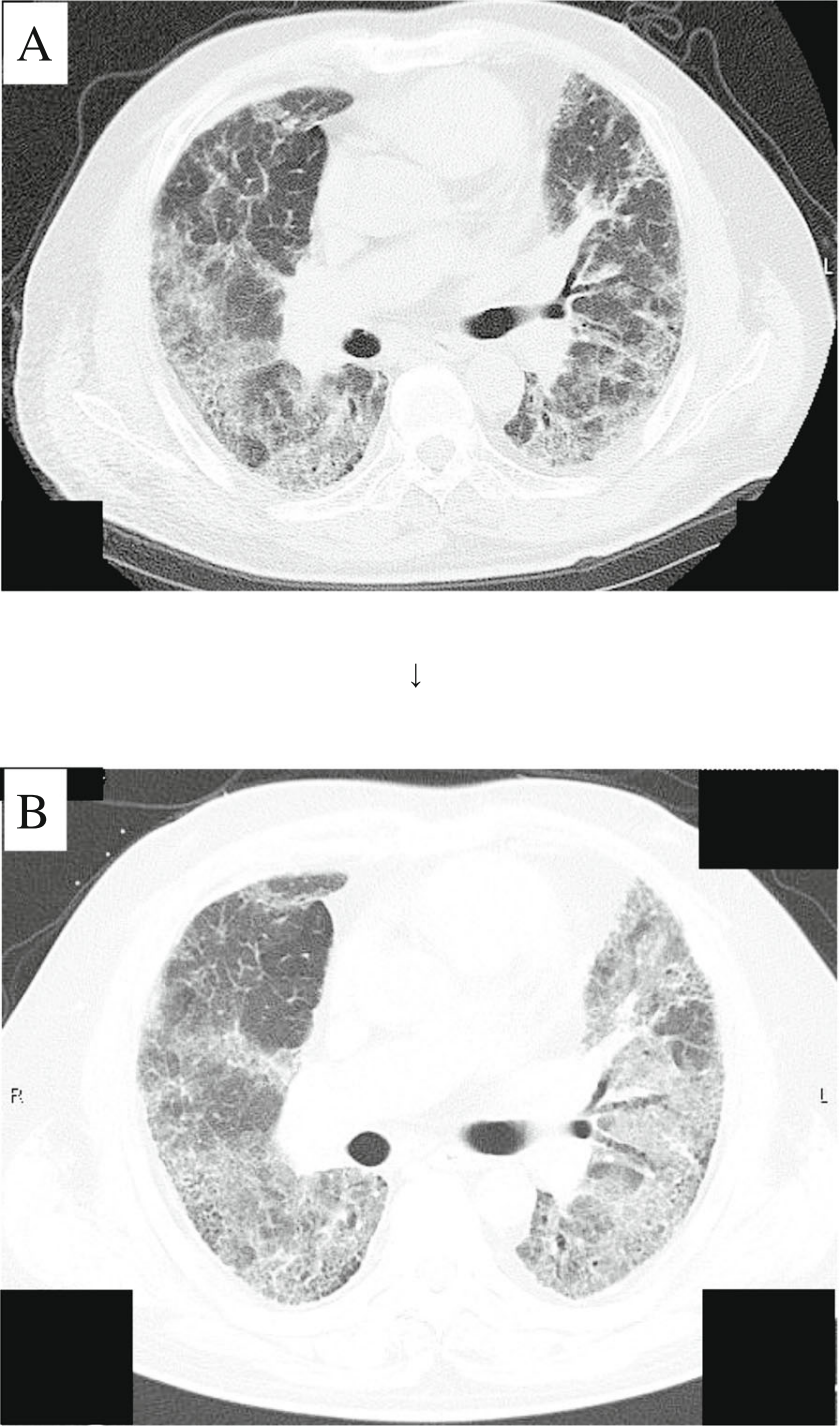
Radiological findings. Chest CT findings for a 57-year-old man. **a** Chest CT findings at 10 months before admission. **b** Chest CT findings at the time of admission showed new ground-glass opacity findings over a broad range of bilateral lung fields

Case 2: The patient was a 57-year-old man who was diagnosed with IPF 5 years earlier and who was currently undergoing oral nintedanib therapy with LTOT. The patient presented at our hospital with the chief complaints of respiratory distress and worsening of cough. Physical examination showed bilateral fine crackles in the lung. Moreover, he exhibited a comparatively poor blood oxygen concentration; new ground-glass opacity was observed over a broad range of bilateral lung fields during CT scanning. He was diagnosed with AE-IPF (Fig. 2). The patient also exhibited a high PT antibody titer (104 EU/mL), according to a measurement taken on day 13 of admission. The patient was able to be discharged to his home with an increased dose of LTOT, following successful clinical treatment.

Neither patient had received any pertussis vaccination since adolescence. As both exhibited a typical usual interstitial pneumonia pattern on high-resolution CT, they were both clinically diagnosed with IPF. No blood test exams or physical findings showed any sign of autoimmune disease. Both patients reported a chronic cough associated with the IPF, but they had been aware of uncontrolled cough deterioration and continuous cough beginning approximately 3 weeks before hospitalization. Neither patient had *Bordetella pertussis* detected from sputum; moreover, PCR analysis was not performed, so the patients did not directly show presence of pathogen*.* Although the typical symptoms of pertussis (e.g., inspiratory whoop) were not observed in either patient, no infectious diseases other than pertussis were detected through sputum culture tests or serum markers. No other causative bacteria were detected in urine antigen tests or sputum culture tests. Moreover, heart failure was not observed in either patient. Both patients were treated with macrolides and broad-spectrum β-lactam antibiotics, accompanied by high-dose corticosteroid therapy. Case 1 involved an initial acute exacerbation and Case 2 involved a recurrent acute exacerbation.

## Discussion and conclusions

The current report described cases that demonstrated infection with pertussis as a cause of AE-IPF. Infection is now considered to be a major causative factor leading to AE-IPF [2]. While various bacteria and viruses have been studied as potential causes of AE-IPF [3, 4], to the best of our knowledge, the cases presented herein constitute the first report in the literature of pertussis as the causative agent of AE-IPF.

The current case report revealed three major findings. The first finding was that consideration of pertussis infection should be noted as part of the differential diagnosis during AE-IPF. Many physicians mistakenly consider pertussis to solely present as a pediatric infection; however, recent publications have shown that pertussis is now common among adults and is often overlooked by internists [5]. Additionally, many adult cases of pertussis do not exhibit typical symptoms [6] and IPF patients often already exhibit persistent dry cough; thus, some physicians may be less likely to initially consider pertussis during the differential diagnosis. These factors may have led to pertussis frequently being overlooked as a potential causative pathogen in cases of AE-IPF.

The second finding was the anticipated efficacy of treatment with macrolide antibiotics. Although further discussion may be necessary, macrolide antibiotic treatment can sometimes reduce the duration or severity of symptoms in pertussis infection [7]. When a patient exhibits AE-IPF, we do not routinely prescribe macrolide antibiotics that are known to be useful for whooping cough. Therefore, our clinical experience may influence antibiotic selection in cases of AE-IPF, because AE-IPF in the current patient may be due to pertussis infection.

Lastly, most bacterial infections of the respiratory tract are not preventable; however, pertussis is one of the few pathogens that is preventable through vaccination. Pertussis vaccination is now recommended in a wide array of developed countries [8]. Vaccination is anticipated to be particularly effective in countries where pertussis vaccinations are not performed after adolescence, as in Japan.

Pertussis infection was diagnosed based on serological testing in our current cases; notably, the serological diagnostic method was validated in multiple previous reports. The major diagnostic criterion of recent or current active pertussis infection is a PT antibody level > 100 EU/mL at any time point; both of our cases met this criterion [9–11]. Previous reports showed that a PT antibody level > 100 EU/mL was comparable to a 4-fold or greater increase in paired serum, or to confirmation of pertussis infection based on positive culture results or polymerase chain reaction testing [12]. In Japan, the cutoff value is established based on the literature [12]: PT antibody titer > 100 EU/mL is used to confirm pertussis infection. In the Japanese infectious disease guidelines, if sputum cultures or Loop-Mediated Isothermal Amplification have not been done or are negative, an increase in antibody titer in the serum has been established as a diagnostic criterion for pertussis; in Japan, therefore, diagnosis by antibody titer is a standard evaluation method. Since a previous study indicated that cultures are unlikely to be positive in adults with more than 3 weeks of coughing, we suspect that a negative culture does not present a problem in the diagnosis of this case [13].

Although widespread adoption of the pertussis vaccine has resulted in a dramatic decrease in the number of affected patients, recent reports of increasing numbers of pertussis cases in various countries around the world are attracting attention [14]. Thus, studies focusing on AE-IPF that may be induced by pertussis are likely to be important in the future.

The mechanism by which pertussis infection induces AE-IPF is currently unclear. The causative *Bordetella pertussis* is known to damage bronchial epithelial cells, thereby inducing inflammatory cytokines and chemokines. PT, adenylate cyclase (ACT), tracheal cytotoxin (TCT), and *Bordetella* dermonecrotic toxin are involved in the pathogenesis of pertussis through the attachment of the bacteria to bronchial mucosal epithelial cells. The presence of TCT induces the production of tumor necrotizing factor alpha, interleukin-6, and IL-1β from bronchial epithelial cells [15]. ACT converts intracellular adenosine triphosphate into cyclic adenosine monophosphate and activates immune response [16]; moreover, ACT plays a role in activating Type 1 T helper (Th1) cells and Th17 cells for further inflammation. In addition to its epithelial damage, *B. pertussis* produces toxins, PT and ACT, that inhibit the phagocytic activity of macrophages in a manner that is distinct, compared with other bacterial pathogens [16, 17].

To our knowledge, this is the first report that acute pertussis infection, a vaccine-preventable and often overlooked infection that is treatable with macrolide, could cause AE-IPF. To epidemiologically investigate the extent to which pertussis is involved in AE-IPF, it is necessary to consider serological and culture examination methods, as well as examination by PCR, which shows high sensitivity. This additional method is needed because the specificity of pertussis is high with serological and culture examination methods, but the corresponding detection rates are low. Further research regarding the relationship between pertussis infection and AE-IPF is critical in the future.

## Abbreviations

ACT: Adenylate cyclase
AE-IPF: Acute exacerbation of idiopathic pulmonary fibrosis
CT: Computed tomography
IPF: Idiopathic pulmonary fibrosis
LTOT: Long-term oxygen therapy
PT: Pertussis toxin
TCT: Tracheal cytotoxin
